# Vitamin D Receptor-Dependent Protective Effect of Moderate Hypoxia in a Mouse Colitis Model

**DOI:** 10.3389/fphys.2022.876890

**Published:** 2022-05-27

**Authors:** Zheng Wang, Hong Yang, Hong Lv, Changzhi Huang, Jiaming Qian

**Affiliations:** ^1^ Department of Gastroenterology, PUMC Hospital, CAMS and PUMC, Beijing, China; ^2^ State Key Laboratory of Molecular Oncology, Cancer Hospital, CAMS and PUMC, Beijing, China

**Keywords:** hypoxia, Vitamin D receptor (VDR), intestinal barrier, DSS-induced colitis, HIF-1α (hypoxia inducible factor-1α)

## Abstract

Although hypoxia is important for maintaining the intestinal barrier, its effect on the barrier during acute colitis and the underlying mechanisms are not fully understood. To explore the influence of hypoxia in dextran sulfate sodium (DSS)-induced colitis mice and the role of hypoxia-inducible factor (HIF) and vitamin D receptor (VDR) in the process. Colitis mice were subjected to hypoxia to detect intestinal barrier function changes. And the mechanisms were explored *in vitro*. First, compared with colitis mice without hypoxia stimulation, those with hypoxia stimulation showed significantly decreased pathological damage and improved permeability of the intestinal barrier. The expression of tight junction proteins (occludin, ZO-1), HIF-1α as well as VDR was up-regulated in colitis mice with hypoxia stimulation. However, in VDR gene knockout (KO)colitis mice, hypoxia treatment showed no protective effect, suggesting the VDR dependency of this effect. Similarly although hypoxia stimulation could enhance the single-layer epithelial transmembrane electrical resistance in DLD-1 and NCM460 cells, these effects disappeared in VDR-knockdown cells. Furthermore, over-expression of HIF-1α in DLD-1 and NCM460 increased the expression of VDR, whereas HIF-1α-knockdown reduced the VDR expression directly. Chromatin immunoprecipitation and luciferase assays confirmed that HIF-1α can bind to the promoter region of the VDR gene under hypoxia. Finally, compared with their wild-type siblings, VDR-KO mice showed reduced abundance of anaerobic bacteria and SCFA-producing bacteria. Hypoxia was protective against DSS-induced colitis, and VDR is instrumental in it. Furthermore, HIF-1α-VDR mediates the effect of hypoxia on the barrier function. Moreover, intestinal flora may be an important link between hypoxia and VDR.

## Introduction

The intestinal mucosal barrier is the first line of defense against a hostile environment within the intestinal lumen, and its integrity is vital for preventing intestinal disorders and systemic diseases. Due to the special blood flow characteristics of the intestine (wherein the arterial blood is rich in nutrients but low in oxygen) and the presence of numerous bacteria, the baseline oxygen partial pressure in the intestine is low (Albenberg et al., 2014; Zheng et al., 2015). However, intestinal epithelial cells are extremely sensitive to reduced blood flow and the consequent tissue hypoxia (Colgan and Taylor, 2010). Thus, intestinal epithelium must have certain mechanisms to regulate barrier function under hypoxia (Glover et al., 2016).

Our previous research on colitis mice suggested that the vitamin D–vitamin D receptor (VDR) pathway has a protective effect against colitis on the intestinal barrier. Hypoxia-inducible factor (HIF), which is a member of the Per-ARNT-Sim family Basic Helix–Loop-Helix (bHLH) transcription factors, can affect the intestinal barrier by regulating several genes responsible for barrier-protective functions (e.g., intestinal trefoil factor, mucin-3, and claudin-1) and relieve animal experimental colitis (Van Welden et al., 2017). The HIF pathway is a classic mechanism that enables cell-specific adaptation of the body to a hypoxic environment, including both long-term or short-term hypoxia. Most studies have reported HIF-1 as beneficial for animal experimental colitis and the intestinal mucosal barrier. For example, HIF-1 gene knockout promoted the inflammatory response in colitis mice, and inhibiting HIF-1 degradation reduced intestinal epithelial cell apoptosis and relieved dextran sulfate sodium (DSS)-induced colitis in mice (Cummins et al., 2008). The influence of hypoxia in DSS-induced colon inflammation has been fully investigated in detail in a previous study (Cosin-Roger et al., 2017). However, few studies have focused on the effects of hypoxic treatment on barrier function during colitis, particularly the regulatory mechanism of VDR on the intestinal mucosal barrier under hypoxia. Herein, we confirmed that 1) hypoxia exerts a barrier protective effect o in a DSS-induced mouse colitis model and VDR is instrumental in it and that 2) the regulatory role of HIF-1α on VDR in protecting the intestinal mucosal barrier. Furthermore, we conducted a preliminary exploration of the influence of VDR deficiency on intestinal flora.

## Materials and Methods

### Ethics Statement

The use of C57BL/6 mice and the study protocol were approved by the Animal Care of Peking Union Medical College (PUMC). All mice were housed in a barrier facility under standard conditions according to the protocols of the PUMC vivarium. All researches were carried out with the approval of the PUMC ethics committee.

### Mouse Colitis Model and Hypoxia Treatment

Herein, 8–10-week-old C57BL/6 mice were 1) given 1.5% DSS in their drinking water for 6 days in the colitis group (*n* = 10), 2) fed in a hypoxic chamber (oxygen content 8%) for 18 h in the hypoxia group (Cosin-Roger et al., 2017) (*n* = 12), which was generally well-tolerated, and 3) not administered either treatment in the control group (*n* = 6). We conducted another experiment of DSS-induced colitis (2.5% DSS for 7 days) treated with Dimethyloxallyl Glycine (DMOG) (Milkiewicz et al., 2004; Cummins et al., 2008) (#sc-200755, Sigma-Aldrich, United States). On days 0, 2, 4, 6, and 8, the DSS + DMOG group was given DMOG i. p. (8 mg in 0.5 ml saline) while DSS colitis group were injected with sterile saline (0.5 ml) at the same intervals (*n* = 5). Control group (Ctrl) receive normal water and sterile saline (*n* = 5).

### Establishment, Identification, and Rearing of Vitamin D Receptor Knockout Mice

Establishment: We used the clustered regularly interspaced short palindromic repeats (CRISPR)/spCas9 technology to obtain VDR knockout (KO) homozygous mice. The conserved functional region Exon3 (Exon3) in the VDR genome structure was selected as the target.

Identification: After weaning, the mice tail or toes were sampled and lysed for polymerase chain reaction (PCR) to obtain the genotype. The PCR primer design strategy and identification method for genotyping were shown in the Supplementary Figure S1.

Rearing and phenotype: Reared in the Animal Laboratory of the Chinese Academy of Medical Sciences, VDR heterozygous mice were bred among close relatives, and VDR-KO mice were used for subsequent experiments. Compared with their wild type (WT) siblings, VDR knockout mice showed no obvious differences before weaning. After weaning, VDR knockout mice gradually showed hair loss, bone thinning, atrophy of skin, and reduced fertility began from 3 to 4 months of age (as showed in Supplementary Figure), which were consistent with previous literature (Zhang et al., 2005).We used high-lactose feed (main ingredients: lactose, 20%; vitamin D, 2.2 IU/g; calcium, 2%; and phosphorus, 1.25%) for routine feeding according to the literature (Zhang et al., 2005), which can prolong the lifetime of VDR knockout mice and alleviate systemic manifestations of vitamin D deficiency, such as rickets and skeletal dysplasia; the mice were specific pathogen-free and exposed to a 12-h/12-h night/day cycle.

### Fluorescein Leakage Experiment

Intestinal barrier permeability was measured using Cellvizio® small animal live confocal fluorescence microscope (Mauna Kea Technologies, France). The azo dye Evans Blue (#E8010, Solarbio, China) was injected through venae angularis to locate the blood vessels. Fluorescein isothiocyanate (FITC)–dextran (70 kD) (#46945, Sigma-Aldrich, United States) was administered through the anus to check the permeability of the intestinal barrier by observing whether it penetrated into the microvessels of the intestinal mucosa. The probe (Z-1800) was advanced 2–3 cm into the anus, and 10 fields of view were observed. The collected image depth was 70 μm; the field of view was 600 × 600 μm; and the laser wavelengths were 488 and 660 nm. Red fluorescence represented blood vessels and green fluorescence represented the presence of FITC–dextran. A mix of red and green fluorescence indicated the leakage of 70-kD FITC–dextran into the microvessels and reflected increased intestinal barrier permeability.

### Evaluation of Mouse Colitis

The mice were subjected to CO_2_ anesthesia and then euthanized. The colon was removed, and the length was measured. Then, the whole colonic “Swiss roll” was embedded and sliced for hematoxylin and eosin (HE) staining, and the pathological score was calculated according to the literature (Zhao et al., 2012).

### Electron Microscope Observation of the Tight Connection Structure

After colon removal, a 1-cm tissue was quickly cut near the anus with a razor blade and immediately put in 2.5% glutaraldehyde solution for subsequent operations. Then, tight junction structure was observed under an electron microscope.

### Mice Fecal Flora Sequencing Analysis

After weaning, the mice were genotyped and confirmed. Then, mice with wild-type VDR ( +, + ) and VDR-KO homozygous VDR (-, -) were housed in separated cages. Feces were collected from each group after 4 weeks of separate housing. Sequencing and data analysis of Miseq (PE300) were performed in the 16S V3–V4 area of the fecal flora.

### Cell Culture and Hypoxic Treatment Conditions

The human colon cancer cell line DLD-1 and naive colon cell line NCM460 were purchased from the American Type Culture Collection (United States) and cultured in RPMI 1640 (#72400047, Gibco, United States) or DMEM(#11995065, Gibco, United States) medium with 10% fetal bovine serum (FCS, Invitrogen, San Giuliano Milanese, Italy). The normal incubator was set to 5% CO_2_ at 37°C, and the hypoxic incubator was set to 94% N_2_, l% O_2_, and 5% CO_2_ at 37°C. The hypoxia group was given 1% O_2_ for 48 h, and the solution was changed once every 24 h. Dimethyloxallyl Glycine (DMOG) (#sc-200755, Sigma-Aldrich, United States) was used at concentrations of 1 mmol/l under normal incubator for 24 h (Heinis et al., 2010).

### Lentiviral Packaging

The following plasmids (#HSH018474-LVRU6GP,# HSH100153-LVRU6GP, #EX-T0096-LV105, GeneCopoeia, MD, United States) were co-transfected into 293 T cells:1) the VDR interference plasmid (sh-VDR) (small hairpin RNA, sh-RNA), 2) the HIF-1α interference plasmid (sh-HIF-1α),3) empty plasmid sh-NC (normal control, NC), 4) HIF-1α overexpression (OE) plasmid (OE- HIF-1α),5) empty vector (EV) and lentivirus packaging plasmids 6) pMD2. G and 7) psPAX2. Cell supernatants were collected at 48 and 72 h to obtain sh-VDR, sh- HIF-1α,sh-NC, OE- HIF-1α and EV viruses. Then, DLD-1 or NCM460 were seeded at 20%–30% density in culture dishes. After 48 h of virus infection, they were screened with 6 μg/ml or 2ug/ml puromycin. After 1 week of continuous culture, stable clones were obtained, and subsequent experiments were carried out.

### Cell Transmembrane Electrical Resistance Experiment

DLD-1 or NCM460 cells in good condition were digested and counted. Then, they were inoculated (100 μL, comprising approximately 1 × 10^5^ cells) in the Transwell chamber. Next, transmembrane resistance of the cells was tested after continuous culture for 1 week.

### Western Blotting

The cells or colonic tissues were fully lysed, and total protein was extracted, which was separated on SDS–PAGE gel and transferred to a membrane. The primary antibodies ZO-1 (#ab96587,Abcam, UK), E-cadherin (#3195S, Cell Signaling Technology, MA, United States), occludin (#ab216327,Abcam, UK), claudin-1 (#A2196,ABclonal, China), Anti-Carbonic Anhydrase 9(CA9) (#ab243660,Abcam, UK), HIF-1α (#ab228649,Abcam, UK) and Histon H_3_ (#4499S, Cell Signaling Technology, MA, United States) were added after blocking nonspecific binding sites with a protein blocker at 4°C for 24 h. Then, they were washed with the Tris–Tween buffer, and secondary antibody was added. This was followed by incubating for 1 h and washing. Then, a chemical luminescent substrate (#A38555, Thermo Fisher Scientific, MA, United States) was added and visualized with a fluorescent chemiluminescence imager. Densitometric analysis of bands was performed using the ImageJ software (version 1.47, National Institutes of Health, NIH, United States).

### Reverse Transcription-Polymerase Chain Reaction

Cell RNA was extracted, and the genomic DNA was removed. Then, cDNA was synthesized by reverse transcription. PCR reaction cycles were as follows: pre-denaturation at 95°C for 30 s, denaturation at 95°C for 10 s, annealing at 60°C for 30 s, and extension at 95°C for 15 s; this was repeated for 45 cycles. The relative expression level was calculated based on the internal reference.

### Chromatin Immunoprecipitation

Chromatin was extracted from DLD-1 cells under normoxia or hypoxia (1% O_2_ for 48 h) separately. Rabbit anti-human HIF-1α antibody and rabbit IgG as a negative control were used to immunoprecipitate chromatin. Immunoprecipitated DNA was analyzed by qPCR using the following sets of primers.

VDR.

Forward: 5ʹ–GCC​ACG​CTG​TAG​CCT​TAG​AT–3ʹ.

Reverse: 5ʹ–GTAGAACCACGGCAGGAAGG–3ʹ

Data are presented as fold enrichment compared to IgG control.

### Luciferase Fluorescein Reporter Gene System

The human VDR promoter was cloned by PCR to generate a VDR promoter reporter gene vector (HPRM30353-PG04, GeneCopoeia, MD, United States). HIF-1α transcription factor plasmid (EX-T0096-M02, GeneCopoeia) and control plasmid (NRG-PG04, GeneCopoeia) were used in this experiment. HT-29 cells were co-transfected with NRG-PG04 + EX-T0096-M02, EX-T0096-M02, and HPRM30353-PG04. After 24 h of culture under normoxia or hypoxia, the cells were lysed and analyzed for luciferase activity using Secrete-Pair™ Gaussia Luciferase Assay Kit (GeneCopoeia).

### Statistical Processing

SPSS 21.0 software was used for statistical processing of the results. T-tests or one-way analysis of variance were used to compare the differences in relative expression between groups. The least significant difference method (equal variance) or Dunnett’s T3 method (uneven variance) was used for multiple-group comparisons; *p* < 0.05 indicated statistically significant difference.

## Results

### Hypoxia Mitigates Dextran Sulfate Sodium-Induced Colitis and Mucosal Barrier Loss in Mice

**FIGURE 1:**
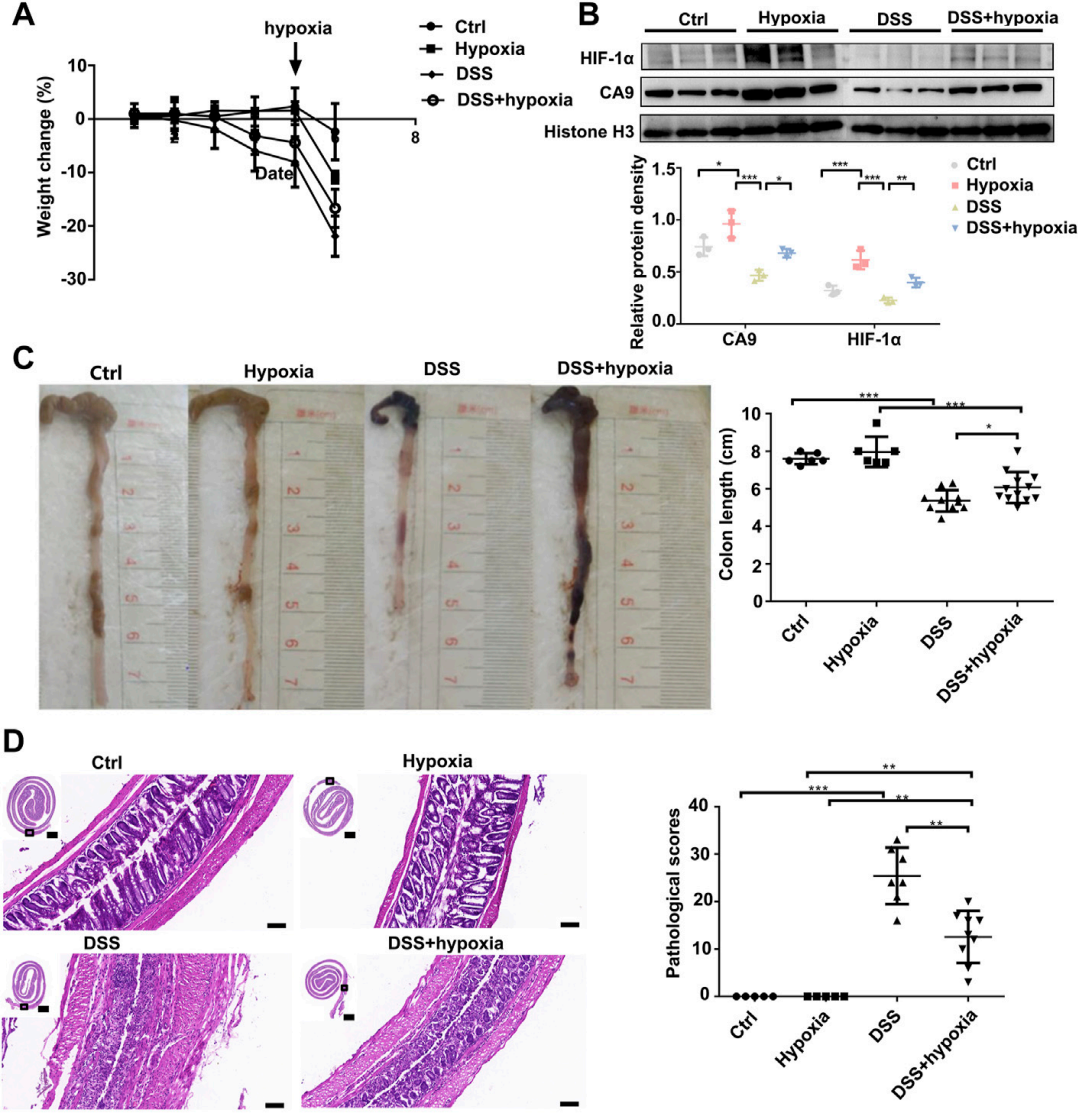
Hypoxia ameliorated DSS-induced colitis in mice. **(A)**: The body weight change of Ctrl, control group; hypoxia, hypoxia-only group; DSS colitis group (DSS), and colitis treated with hypoxia group (DSS + hypoxia); on day 5, the hypoxia group was given 1% O_2_ stimulation. **(B)**: HIF-1α and CA9 (Carbonate dehydratase 9) expression by Western blotting in each group. **(C)**: Length of the colon at the end of the experiment in each group. (***P < 0.001, *P < 0.05). **(D)**: The HE staining of the colon and their pathological scores in each group. (***P < 0.001, **P < 0.01). Bars = 2000 μm or 100 μm.

**FIGURE 2:**
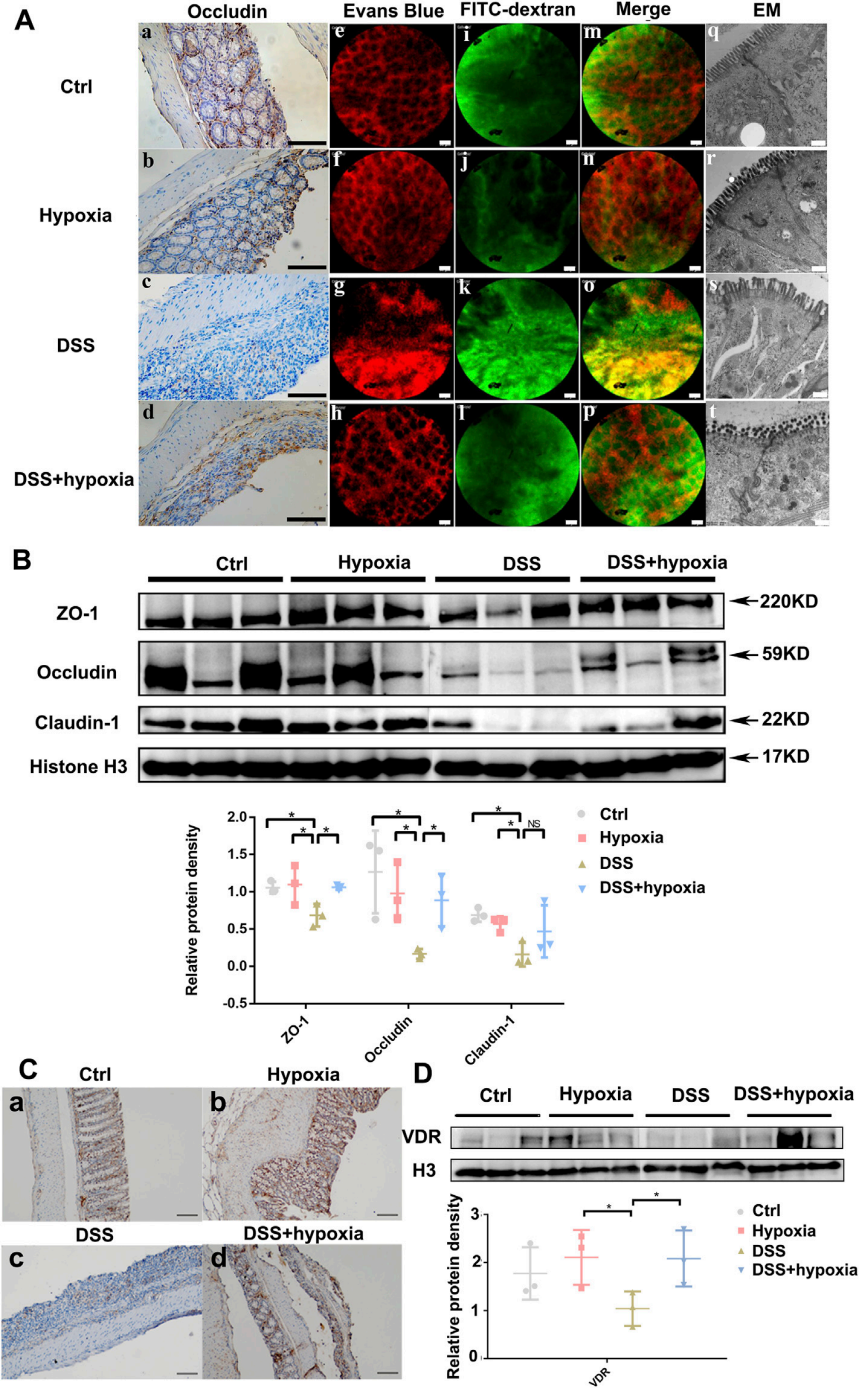
The impact of hypoxia treatment on the intestinal mucosal barrier in mice with DSS-induced colitis. **(A)** (a–d): Immunohistochemistry staining of occludin in each group. Bars = 100 μm. **(A)** (e–p): Intravital fluorescein penetration experiments of the mice intestinal mucosa near the anus in each group. Blood vessels (red) were blurred, and 70–kD FITC–dextran (green) was visible in the impaired intestinal mucosa in the DSS group [**(A)**. g, k, and o], which was improved in the DSS + hypoxia group [**(A)**. h, l, and p]. Bars = 50 μm. **(A)** (q–t): The tight junction structure under electron microscope of each group. Bars = 500 nm. **(B)**: Tight junction protein expression (including ZO-1, occludin and claudin-1) by Western blotting in each group. **(C)** (a-d): Representative pictures of VDR protein expression by immunohistochemistry in each group. Bars = 100 μm. **(D)**: VDR protein expression by Western blotting analysis in each group. All experiments were repeated three times.

As shown in Figure 1, compared with the DSS group (DSS), the weight loss rate slowed down in the DSS + hypoxia group (Figure 1A), and colon inflammation in the DSS + hypoxia group was lesser, which translated into improved colon length (Figure 1C) and better pathology score (Figure 1D). The expression of HIF-1α and CA9 in the intestinal mucosal were increased after hypoxia treatment (Figure 1B). The destruction of the tight junction structure under electron microscope was mitigated in the DSS + hypoxia group when compared with the DSS group (Figure 2A Figure 2s,t). In addition, IHC also showed increased expression of occludin in the DSS + hypoxia group. Fluorescein leakage experiment *in vivo* showed blurred blood vessel texture and fused FITC-70 kD (green fluorescein) into blood vessels in the DSS group [Figure 2A (e,i,m,g,k and o)], while the leakage of FITC-70 kD in the capillaries was reduced in the DSS + hypoxia group [Figure 2A (g,k,o,h,l and p)]. Western blotting showed that the expression of tight junction proteins (e.g. ZO-1 and occludin) was higher in the DSS + hypoxia group than in the DSS group (Figure 2B). Furthermore, VDR was elevated in the DSS + hypoxia group compared with the DSS group (Figure 2C).

VDR was indispensable for mucosal barrier function under hypoxia in VDR-KO mice with DSS-induced colitis.

**FIGURE 3:**
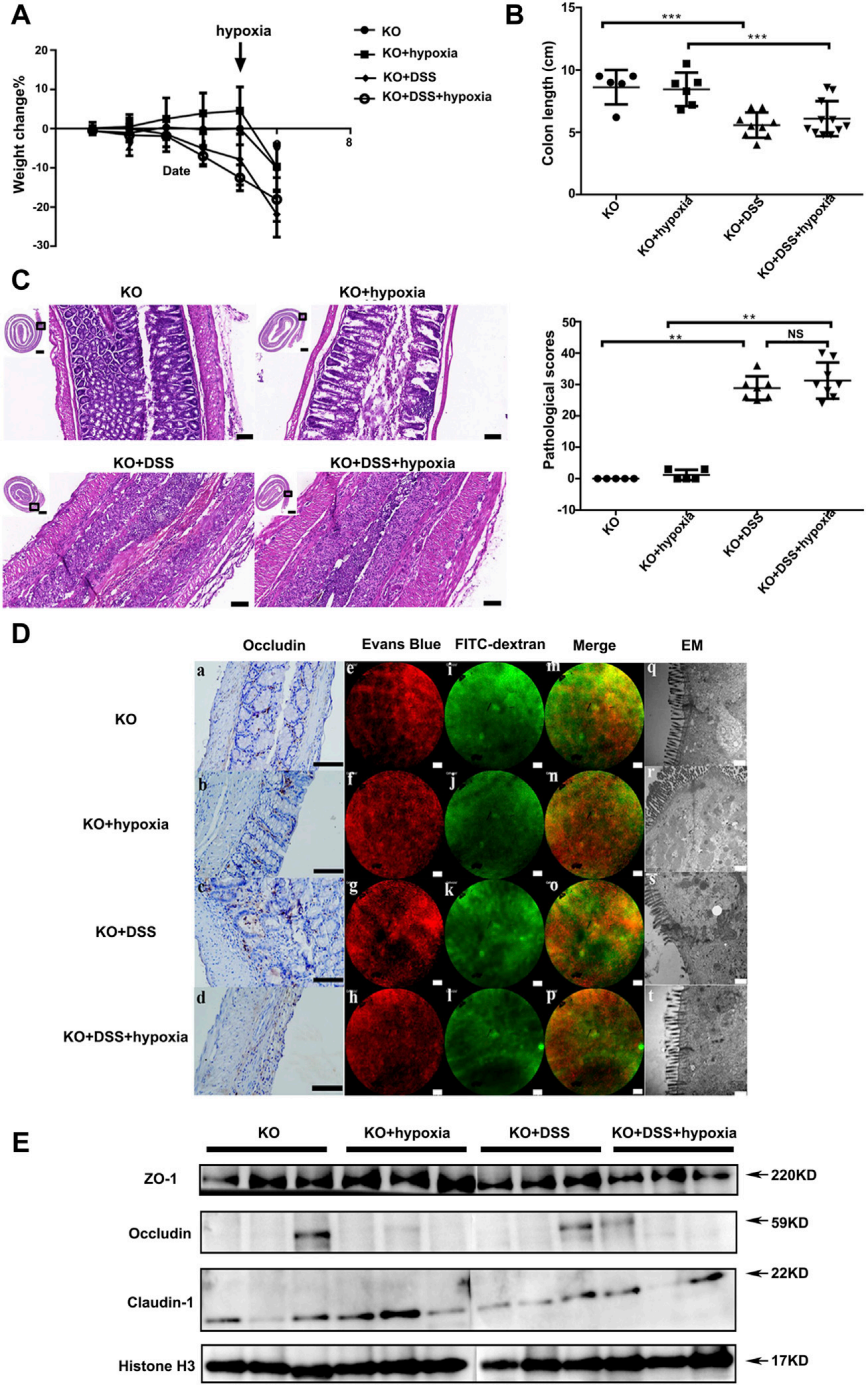
VDR was indispensable for mucosal barrier function under hypoxia in VDR knockout mic. **(A)**: The percentage change of body weight in VDR deficiency mice: VDR knockout mice (VDR-KO), hypoxia treatment group (VDR-KO + hypoxia), DSS colitis in VDR-KO mice (VDR-KO + DSS), and colitis treated with hypoxia group (KO + DSS + hypoxia). **(B)**: Colon length at the end of the experiment in each group. **(C)**: Pathological manifestations of the mouse colon and their pathological scores (n ≥ 5, ***P < 0.001, **P < 0.01; NS: no significant difference). Bars = 2000 μm or 100 μm. **(D)** (a–d): Occludin expression by IHC in VDR-KO, VDR-KO + hypoxia, VDR-KO + DSS, and KO + DSS+ hypoxia groups. Bars = 100 μm. **(D)** (e–p): Regarding intravital fluorescein penetration experiments in each group, no differences were detected between VDR-KO + DSS group [**(D)** g, k, and o) and KO + DSS + hypoxia group [**(D)** h, l, and p]. Bars = 50 μm. **(D)** (q–t) The tight junction structure under an electron microscope in each group. Bars = 500 nm. **(E)**. Expression of tight junction proteins (ZO-1,occludin and claudin-1) by Western blotting in each group. The experiment was repeated three times.

We used VDR-KO mice to further investigate if the absence of VDR abolishes the effect of hypoxia on acute colitis. As shown in Figure 3, compared with the VDR-KO control group, the VDR-KO colitis group (KO + DSS) showed obvious weight loss (Figure 3A), shortened colon (Figure 3B). Histologically, severe inflammation and destroyed colonic epithelium could be seen after DSS treatment (Figure 3C). Previously, we proved that hypoxia can alleviate colitis and intestinal mucosal barrier damage in DSS mice; however, after VDR knockout, hypoxia treatment could not reduce the rate of weight loss (Figure 3A), improve the colon length (Figure 3B), and improve the pathological score (Figure 3C) in VDR-KO colitis mice. Moreover, there were no differences in occludin expression between the KO + DSS group and KO + DSS + hypoxia group (Figure 3D (c,d) and Figure 3E). Under an electron microscope, the VDR-KO group and KO + hypoxia group showed incomplete tight junction structure and slightly blurred edges, which reflected the loss of the barrier structure [Figure 3D (q,r,s and t)]. We performed an *in vivo* intestinal fluorescein leakage test and identified that the barrier function of the above two groups was also impaired, and some FITC-70 kD (green fluorescein) had penetrated into the blood vessels (red fluorescein) (Figure 3D). After DSS treatment, the red blood vessel texture disappeared, and the green fluorescence and red fluorescence almost completely merged, indicating that the barrier function was lost and FITC had completely infiltrated the blood vessels, and the above situation did not improve after hypoxia treatment (Figure 3D).

### Vitamin D Receptor Served as a Key Factor in Hypoxia-Induced Mucosal Barrier Protection

#### Vitamin D Receptor Was Indispensable for Protecting Single-Layer Epithelial Barrier *in vitro*

**FIGURE 4:**
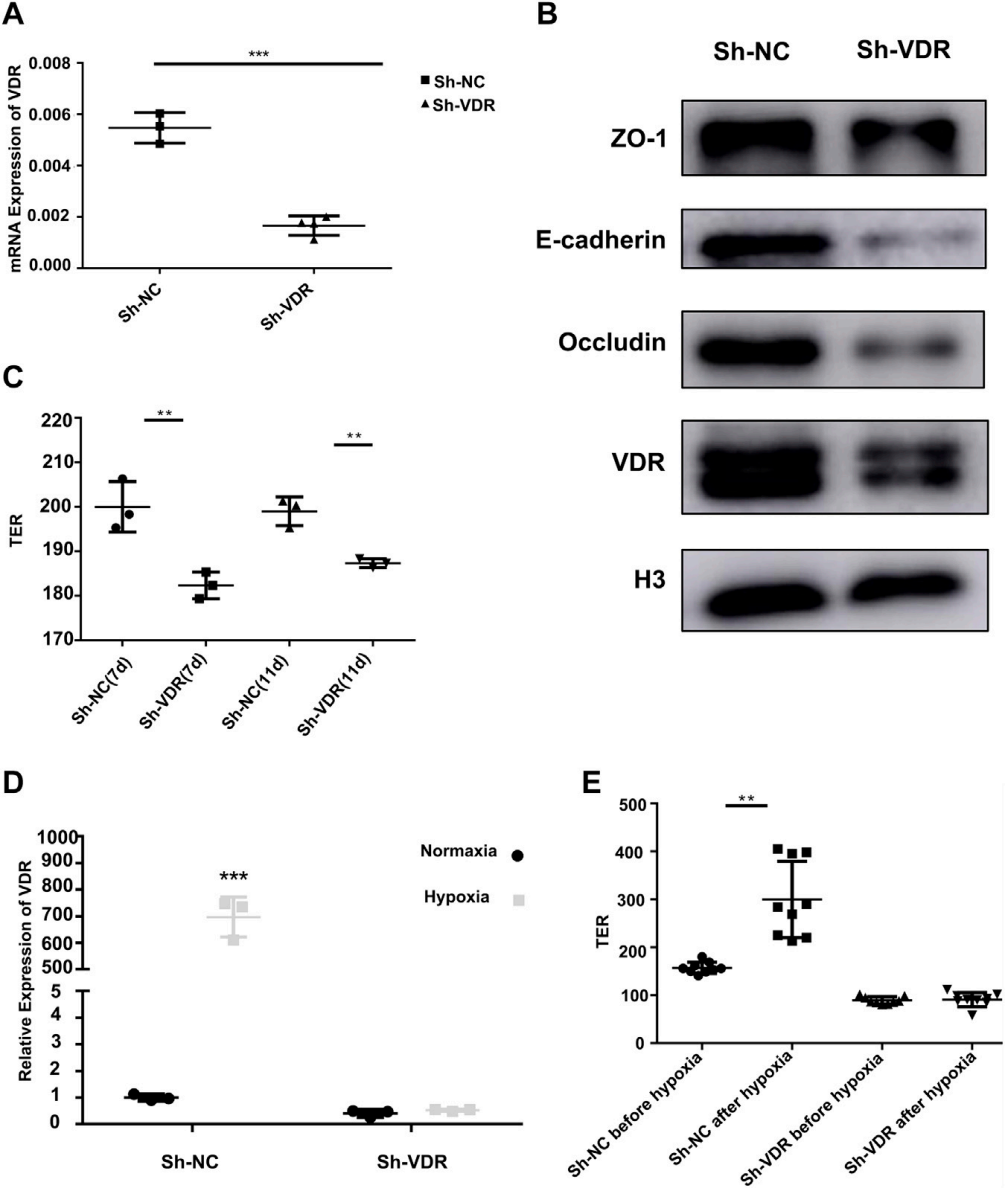
VDR protecting mucosal barrier protein and barrier function *in vitro* **(A)**: VDR mRNA expression by RT-PCR in DLD-1 sh-VDR cells and their control sh-NC cells (***P < 0.001). **(B)**: The expression of the mucosal barrier proteins ZO-1, E-cadherin, occludin, and VDR by Western blotting in DLD-1 sh-VDR cells. **(C)**: TER values of DLD-1 sh-NC and sh-VDR cells after continuous culture for 7 days and 11 days (*n* = 3;**P < 0.01). **(D)**: VDR mRNA expression in sh-NC and sh-VDR cells under 1% O_2_ for 48 h. (***P < 0.001). **(E)**: The TER value of sh-VDR and sh-NC cells before and after hypoxic treatment (**P < 0.01). The experiments were repeated at least three times.

We constructed stable VDR-knockdown cell lines of DLD-1 and NCM460 using lentiviral plasmids (Figure 4A and Supplementary Figure S2A). The expressions of the mucosal adhesion protein E-cadherin and the tight junction proteins ZO-1 and occludin were significantly reduced after VDR-knockdown (Figure 4B and Supplementary Figure S2A). Then, the transmembrane electrical resistance of DLD-1 or NCM460 sh-NC and sh-VDR cells was detected after the cells were continuously cultured in a transwell capsule for 7 days and 11 days. As shown in Figure 4C and Supplementary Figure S2B, transmembrane electrical resistance (TER) values of DLD-1 sh-VDR cells were significantly lower than those of DLD-1 sh-NC cells (*n* = 3, 200.0 ± 5.69 vs 182.3 ± 3.00, *p* = 0.009; *n* = 3, 199.0 ± 3.21 vs 187.3 ± 1.00, *p* = 0.004). Similarly, TER values of NCM460 sh-VDR cells were significantly lower than those of NCM460 sh-NC cells (*n* = 3, 97.2 ± 1.37 vs 85.3 ± 2.52, *p* = 0.002).

### Vitamin D Receptor Was Crucial for Protecting Mucosal Barrier Function Under Hypoxia *in vitro*

To further confirm the role of VDR on the intestinal epithelial barrier under hypoxia, we tested VDR mRNA expression in DLD-1 sh-VDR cells and their control sh-NC cells under 1% O_2_ incubation for 48 h. The results showed that hypoxia treatment significantly increased VDR expression in DLD-1 sh-NC cells, as shown in Figure 4D. However, hypoxic stimulation could not induce VDR mRNA expression after VDR knockdown. Furthermore, as showed in Figure 4, in DLD-1 sh-NC cells, hypoxia treatment increased transmembrane electrical resistance value (*n* = 9, 157.33 ± 3.88 vs 299.89 ± 26.50, *p* = 0.003); however, there was no difference in the TER value after 1% O_2_ incubation in sh-VDR cells (*n* = 9, 89.44 ± 2.49 vs 90.89 ± 4.95, *p* = 1.0) (Figure 4E). Accordingly, in NCM460 sh-NC cells, hypoxia treatment increased TER value (*n* = 6, 93.67 ± 1.21 vs 105.0 ± 3.46, *p* < 0.001); however, there was no difference in the TER value after 1% O_2_ incubation in sh-VDR cells (*n* = 6, 81.5 ± 4.32 vs 85.67 ± 1.86, *p* = 0.15), as shown in Supplementary Figure S2C.

### Hypoxia Impacts on Vitamin D Receptor Which Was Mediated by HIF-1α

**FIGURE 5:**
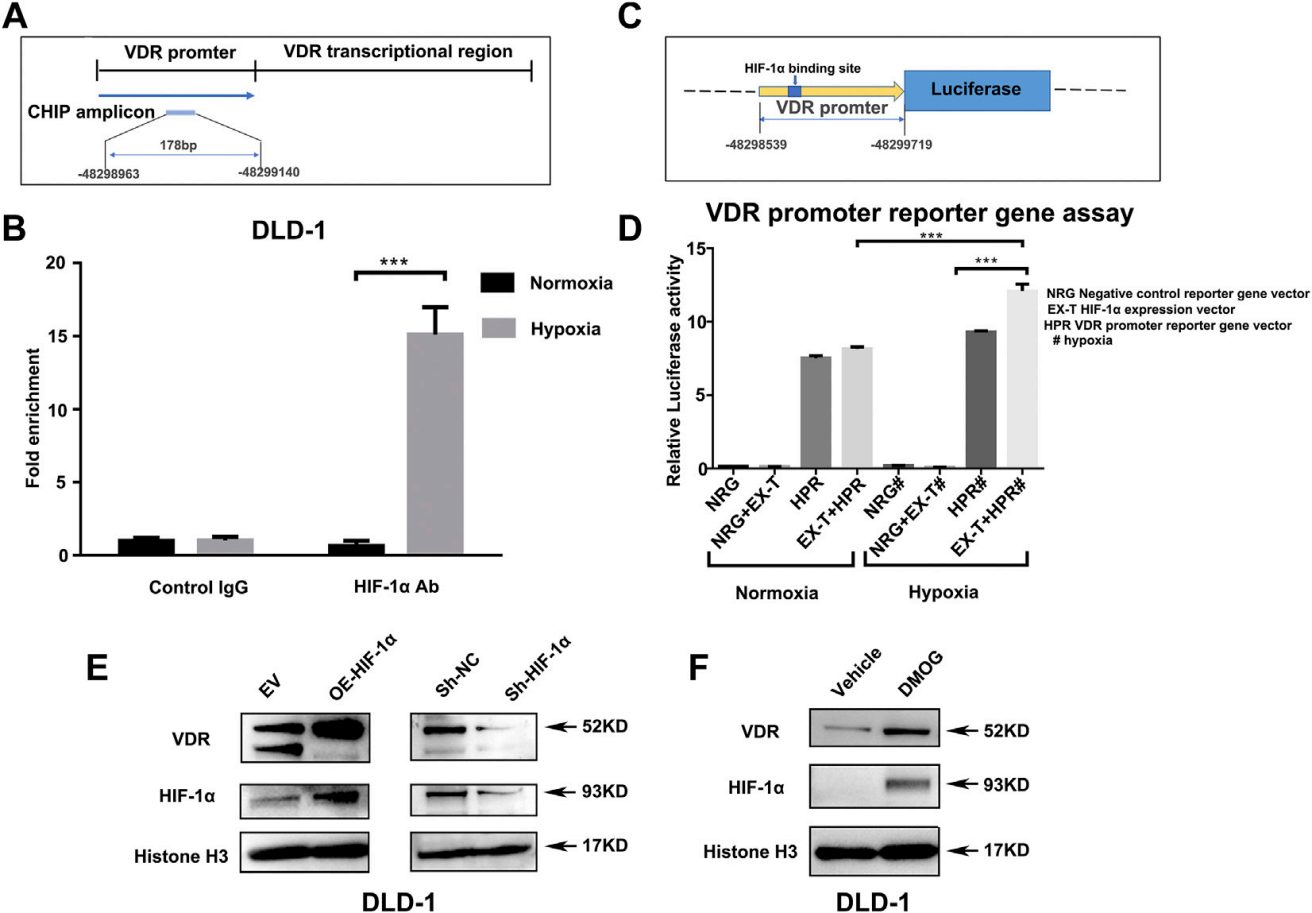
HIF-1α regulates the expression of VDR directly by binding to its promoter region. **(A)**: Schematic presentation of VDR DNA sequences used for Chip assay. **(B)**: Chromatin immunoprecipitation analysis showing the enrichment of VDR DNA expression in the promoter region bound by the HIF-1α antibody in comparison with the IgG negative control. Data are represented as mean ± standard error of mean (***P < 0.001). VDR levels from three independent experiments performed using DLD-1. **(C)**: Schematic presentation of VDR promoter reporter plasmid containing the HIF-1α binding site. **(D)**: Luciferase activity in 293T cells transfected with a negative control reporter gene vector (NRG), VDR promoter reporter gene vector HPRM30353-PG04 (HRP), or HIF-1α expression vector EX-T0096-M02 (EX-T) separately (NRG or HPR) or together (NRG + EX-T or EX-T + HPR) under normoxia and hypoxia (***P < 0.001, # represented hypoxia). **(E)**: Expression of VDR after HIF-1α over-expression (OE) and HIF-1α knock-down using lentiviral plasmids in DLD-1 compared with their control [empty vector (EV) or sh-NC] separately. **(F)**: Expression of VDR after DMOG 1mM treated for 24 h in DLD-1.

Next, we confirmed the interaction between hypoxia and VDR. The Transcription Factor Affinity Prediction software sTRAP (available at http://trap.molgen.mpg.de/cgi-bin/trap_two_seq_form.cgi) suggested the presence of HIF-binding sites in the VDR promoter region. chromatin immunoprecipitation (CHIP) showed (Figures 5A,B) that the enrichment ratio of the hypoxia group was significantly higher than that of the control group, suggesting that HIF-1α can bind to the VDR promoter and mediate downstream signal transcription. To investigate whether the activation of VDR by hypoxia is associated with HIF-1α, the VDR promoter reporter gene vector (HPRM30353-PG04) was co-transfected with the HIF-1α expression vector (EX-T0096-M02) into 293 T cells (Figure 5C). The luciferase assay showed increased luciferase activity with this co-transfection when compared with single transfection of HPRM30353-PG04 (12.065 ± 0.841 vs 9.293 ± 0.166, *p* < 0.0001; lanes seven and eight of Figure 5D). Moreover, co-transfection of HPRM30353-PG04 and EX-T0096-M02 presented better luciferase activity under hypoxia than under normoxia (12.065 ± 0.841 vs 8.144 ± 0.233, *p* < 0.0001; lanes four and eight of Figure 5B), which was attributed to the destabilization of HIF-1α in normoxia. In addition, we established HIF-1α-knockdown (Sh-HIF-1α) and HIF-1α over-expression (OE-HIF-1α) cell lines of DLD-1 using lentiviral plasmids. As shown in Figure 5C, over-expression of HIF-1α in DLD-1 can increase the expression of VDR, whereas HIF-1α-knockdown reduces the expression of VDR directly. Moreover we used DMOG to further investigate its effect on HIF-1α and VDR. The results showed that over-expression of HIF-1α by Dimethyloxallyl Glycine (DMOG) in NCM460 and DLD-1 increased the expression of VDR (as in Figure 5D and Supplementary Figure S2D). While HIF-1α-knockdown by lentiviral plasmids reduced the VDR expression in NCM460(as in Supplementary Figure S2E). Furthermore, we conducted another experiment of DSS-induced colitis treated with DMOG. Compared with the DSS group (DSS), the weight loss rate tended to slowed down in the DSS + DMOG group (Supplementary Figure S3A), and colon inflammation in the DSS + DMOG group was lesser, which translated into improved colon length (Supplementary Figures S3B, SC) and better pathology scores (Supplementary Figures S3D, SE). The expression of VDR in the intestinal mucosal tendeded to increase after DMOG treatment (Supplementary Figure S3F).

### Intestinal Flora Connected the Hypoxic Environment With Vitamin D Receptor

**FIGURE 6:**
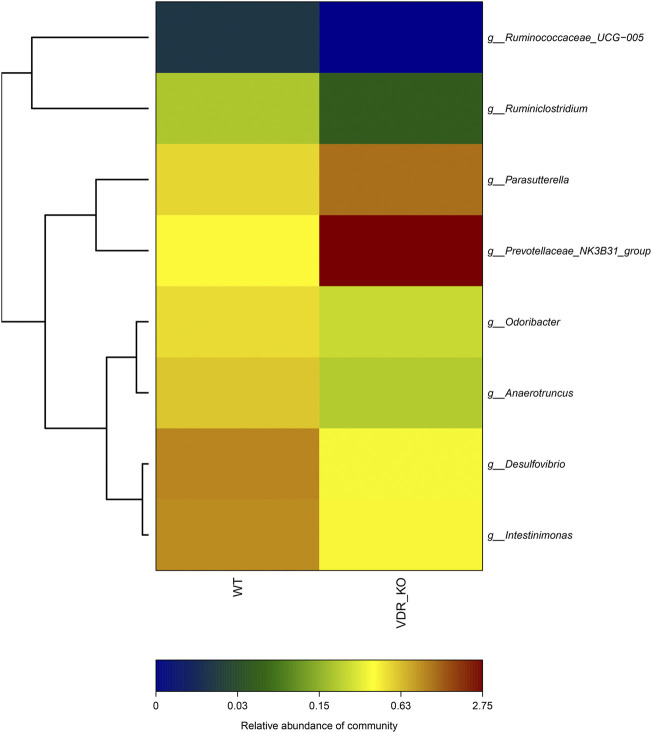
16S Analysis of VDR-KO mouse fecal flora. showed the species and relative difference of wild-type control group (*n* = 5) and VDR-KO group (*n* = 5). The color of the square reflects the relative content of a specific genus in the fecal flora. Red represents high abundance, while closer to dark blue represents less abundance.

Previously, we demonstrated that the environment (hypoxia) can affect gene expression. Since many studies have confirmed that genes interact with the environment, VDR is very likely to affect aspects of the intestinal environment, such as intestinal flora. To minimize the interference factors, we used siblings of VDR-KO mice (*n* = 5) and wild-type mice (*n* = 5) to identify the changes in the fecal flora of VDR-KO mice. The results showed that six bacteria species were reduced in the feces of VDR-KO mice (Figure 6). These species comprised *Desulfovibrio* spp. and *Odoribacter* spp., which are obligate anaerobic bacteria, *Ruminiclostridium* spp., which are related to fiber degradation and can produce short-chain fatty acids (SCFAs), and *Anaerotruncus* spp. and Ruminococcaceae spp*.,* which belong to the family *Clostridium*, and can also produce butyrate, acetic acid, and other SCFAs. *Intestinimonas* spp. is known for its unique metabolic features of producing butyrate from both sugars and amino acids. Other two bacteria species that were increased in the feces of VDR-KO mice were *Parasutterella* spp*.* and Prevotellaceae spp*. Parasutterella* spp. has recently been found to be related with chronic intestinal inflammation in patients with irritable bowel syndrome (Chen et al., 2018). It shows that the lack of the VDR gene affects the number and composition of intestinal flora and reduces the abundance of anaerobic bacteria and SCFA-producing bacteria.

## Discussion

In this study, we found that hypoxia treatment had a protective effect on the intestinal barrier of mice with DSS-induced colitis and that VDR played a key role in it. Subsequently, we confirmed that HIF-1α mediated the effect of hypoxic environment on VDR signaling pathways and further understood the physiological mechanism of the mucous barrier regulation under hypoxia. Finally, we proposed that gut flora may be involved in the interaction between low-oxygen environment and VDR.

The blood vessels in the intestine were rich in nutrients but had relatively low oxygen content. As the blood flowed from the base to the top of the intestinal villi and then back to the venules, the oxygen content would decrease gradually, thus causing physiological hypoxia (Allam-Ndoul et al., 2020). Accumulated evidence shows that physiological hypoxia is beneficial to maintaining intestinal homeostasis (Van Welden et al., 2017). Therefore, the regulation of intestinal barrier function under hypoxia is highly significant to the body’s defense against external aggression and for the prevention of diseases. Our previous studies confirmed that VDR has a regulatory effect on barrier proteins (Supplementary Figure S1); however, its effect under hypoxia has not been reported yet. First, we used wild-type mice and VDR-KO mice to test the effect of hypoxia on the intestinal barrier function at the animal level with an aim to preliminarily explore the influence of hypoxia on colitis mice and their intestinal mucosal barrier as well as the role of VDR in it. The results showed that compared with mice with DSS-induced colitis without hypoxia stimulation, those subjected to hypoxic treatment showed improved colon length, better pathological damage scores, improved mucosa permeability, and improved tight junction ultrastructure. However, hypoxia treatment in VDR-KO DSS mice could not improve colon inflammation and intestinal barrier damage, suggesting that this effect is dependent on VDR. Taken together, hypoxia may mediate the protective effect against acute mouse colitis through VDR.

HIF is instrumental in inducing cellular adaptive responses under hypoxia (Robrahn et al., 2020). HIF comprises an α subunit (divided into HIF-1α, HIF-2α, and HIF-3α) and a β subunit (HIF-1β). Although some studies hold opposing views on the role of HIF in colon inflammation (Xue et al., 2021), it has been well acknowledged that the protective role HIF signaling in colon homeostasis is primarily dependent on HIF-1α (Karhausen et al., 2004), whereas the pro-inflammatory phenotype observed was mainly due to HIF-2α signaling (Shah et al., 2008). HIF-1α was the main regulator of the hypoxia signal, and it was quickly degraded by ubiquitination under normoxia. Recently, a well-designed clinical trial supported the full therapeutic potential of a HIF-1α stabilizer (GB004) for the treatment of active ulcerative colitis (Danese et al., 2022). We previously reported increased expression of the VDR protein and the barrier proteins E-cadherin and claudin-1 in the colon cell line DLD-1 under hypoxia (Wang et al., 2019). To further explore the mechanisms underlying these changes caused by hypoxia, we used a VDR knock down cell line *in vitro* hypoxia experiments and found that hypoxia stimulation increased the expression of VDR mRNA levels in colon cells and enhanced transmembrane resistance of colon epithelial cells. Through bioinformatics predictive analysis, we predicted that the VDR promoter region has a potential binding site for HIF-1α. In addition, the over-expression of HIF-1α in naïve colon cell line NCM460 and DLD-1 cells by DMOG increased the expression of VDR, whereas HIF-1α-knockdown reduced the expression of VDR directly. Subsequent CHIP experiments confirmed that HIF-1α can bind to the promoter region of the VDR gene under hypoxia. The luciferase experiment verified that their combination can activate VDR and induce the downstream transcription. In this study, we confirmed the direct benefits of moderate hypoxia treatment on DSS-induced colitis, and to our knowledge, we are the first to detect and report an interaction between HIF-1α and VDR, wherein HIF-1α binds to the promoter region of VDR under hypoxia and enhances VDR-mediated mucosal barrier protection. Our study further verified the therapeutic mechanism of HIF-1α stabilizer in patients with inflammatory bowel disease.

It is well known that intestinal flora is instrumental in maintaining intestinal homeostasis. Recent studies have shown that bacterial metabolites, such as SCFAs, are particularly important part in the process (Koh et al., 2016). Fecal 16S RNA sequencing analysis of VDR-KO mice (VDR −/−) and their littermate wild-type mice (VDR +/+) showed that some SCFA-producing bacteria (*Ruminiclostridium* spp. and *Anaerotruncus* spp.) were significantly reduced in VDR-KO mice. Several SCFAs, including butyrate, propionate, and acetic acid, were produced by the bacteria in the intestine through the breakdown of cellulose, and these can be directly taken up and used by the intestinal epithelial cells to promote the integrity of the intestinal mucosal barrier (Kelly et al., 2015). Among these SCFAs, butyrate can increase the oxygen consumption of the intestinal epithelium and can help create a low-oxygen metabolic environment which can be perceived by epithelial cells. Studies have suggested that the abovementioned mechanism can increase the stability of HIF-1, which is of great significance to maintain the homeostasis of the intestinal mucosa (Glover et al., 2016). In addition, we also identified that the content of some obligate anaerobes (e.g., *Desulfovibrio* spp., *Odoribacter* spp., and *Ruminiclostridium* spp.) was also significantly reduced in VDR-KO mice. These results suggested that VDR KO may change the hypoxic environment in the gut, making it impossible for obligate anaerobic bacteria to colonize the intestine and exert their physiological effects. These changes in the intestinal tract of VDR-KO mice suggest that the state of VDR affects the microbial composition of the intestinal tract, consequently proving that VDR interacts with the intestinal microenvironment. The intestinal environment, such as hypoxia, can regulate the expression of VDR through HIF-1α; conversely, the state of VDR can also affect the intestinal environment through the intestinal flora.

We herein observed the symptoms of colitis and changes in intestinal barrier function under hypoxia and finally explored the underlying mechanisms associated with the colon cell line. Our study revealed the beneficial effects of moderate hypoxia in mice with DSS-induced colitis and the key role of VDR in it. Our study also proved that HIF-1α mediates the effect of hypoxia on the VDR signaling pathway. Finally, our work suggested that the intestinal flora is an important link between hypoxic environment and VDR.

This study had certain limitations. First, the effects of systemic knockout cannot be overlooked with the use of VDR-KO mice. Thus, we fed the hybrid mice 20% high lactose and vitamin D from the stage of pregnancy, which can reduce the impact of knockout on growth and development to a certain extent. After weaning, VDR-KO mice and wild-type mice had similar phenotypes, and the general form and pathological form of the whole colon showed no significant difference between the mice (Supplementary Figure S1). Next, we admitted that whole-body hypoxia treatment may cause some potential side effects for the mice, such as reduced activity and eating in the hypoxic chamber. However, live local hypoxia treatment is still under research. So we conducted an alternative experiment of DSS-induced colitis treated with DMOG, which is a cell permeable and competitive inhibitor of HIF-PH, and thus results in HIF-1α stabilization and accmulation *in vitro* and *in vivo* (Milkiewicz et al., 2004; Cummins et al., 2008; Heinis et al., 2010). The results showed that compared with the DSS group (DSS), the colitis in the DSS + DMOG group was relieved and the expression of VDR in the intestinal mucosal tended to increase after DMOG treatment (Supplementary Figure S3). This study was significant in that it established the significance of VDR in protecting the intestinal barrier under hypoxic intestinal microenvironment. Furthermore, we also confirmed the internal mechanism of HIF-1α-regulated VDR expression, which provides a basis for us to further explore the mechanism of the action of VDR in diseases associated with a low-oxygen state (e.g., ischemic bowel disease and colon cancer) or intestinal barrier damage (e.g., inflammatory bowel disease).

## Data Availability

The original contributions presented in the study are included in the article/Supplementary Material, further inquiries can be directed to the corresponding authors.
