# Supplementary material for: Vitamin D Receptor-Dependent Protective Effect of Moderate Hypoxia in a Mouse Colitis Model
Source: Front Physiol. 2022 May 27;13:876890. doi: 10.3389/fphys.2022.876890 (PMC9195869; doi:10.3389/fphys.2022.876890)
Supplement: Supplementary file 1 [file Image1.pdf]

Supplementary Material

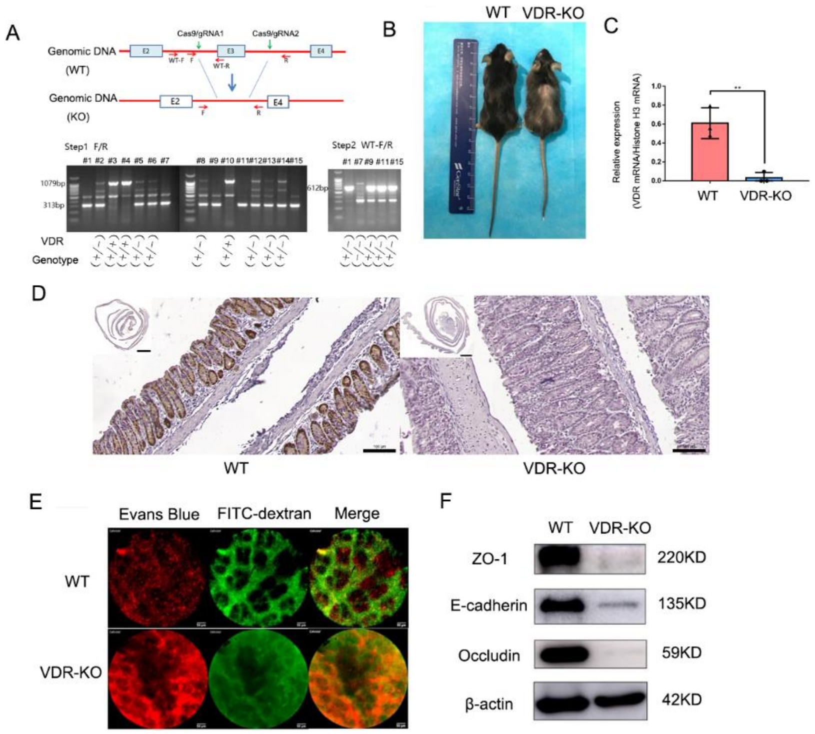

Supplementary Figure1. Characteristics of VDR knock-out mice

Supplementary Figure1A: showed the PCR primer design strategy for genotyping in VDR-KO mice. Clustered Regularly Interspaced short Palindromic Repeats (CRISPR) was used to delete the protein coding region of the Exon3 in the VDR genome structure. First pair of primers (F/R) : The length of PCR fragment was 1079bp for wild type, 313bp for homozygous knockout mice, and 1079bp plus 313bp or only 313bp for heterozygous (because the long fragment was at a disadvantage in competitive synthesis with short fragment, which could not be clearly displayed on the gel. Therefore, a second pair of primers is needed for further identification). The second pair of primers (WT-F/R) : The length of PCR fragment was 612bp for wild type or heterozygous, while homozygous knockout mice do not have any product.

Supplementary Figure1B showed the phenotype of VDR-KO mice at 8-week. compared with their wild type (WT) siblings, VDR knockout mice showed no obvious differences before

weaning. After weaning, VDR knockout mice gradually showed hair loss, bone thinning, atrophy of skin, and reduced fertility began from 3–4 months of age. Supplementary Figure1(C-D) showed the expression of VDR mRNA and protein in VDR-KO mice by qRT-PCR and IHC. Supplementary Figure1E Intravital fluorescein penetration experiments of the mice intestinal mucosa near the anus in VDR-KO mice. Compared with WT mice, VDR knockout mice had abnormal intestinal permeability as 70kD FITC–dextran (green) was slightly visible in the blood vessels (red) in the VDR-KO mice. Supplementary Figure1F :The expression of the barrier proteins (including ZO-1, E-cadherin, Occludin) in VDR KO mice by Western-blot .

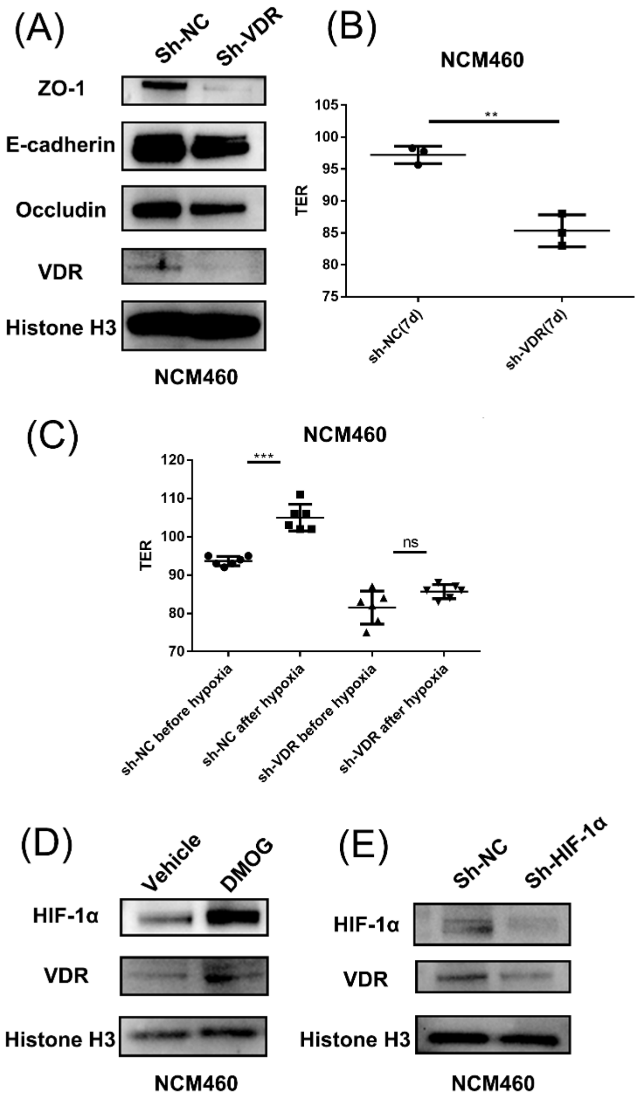

Supplementary Figure2. VDR protecting mucosal barrier protein and barrier function in NCM460

Supplementary Figure2 A: The expression of the mucosal barrier proteins ZO-1, E-cadherin, occludin, and VDR by Western blotting in sh-VDR cells of NCM460. Supplementary Figure2B: TER values of NCM460 sh-NC and sh-VDR cells after continuous culture for 7 days (n = 3;***P* < 0.01). Supplementary Figure2C: The TER value of sh-VDR and sh-NC cells before and after hypoxic treatment for 48h(****P* < 0.001). Supplementary Figure2D: Expression of VDR after DMOG(1mM) treated for 24h in NCM460. Supplementary Figure2F: Expression of VDR after HIF-1α knock-down using lentiviral plasmids (sh-HIF-1α) in NCM460 compared with the control (sh-NC).

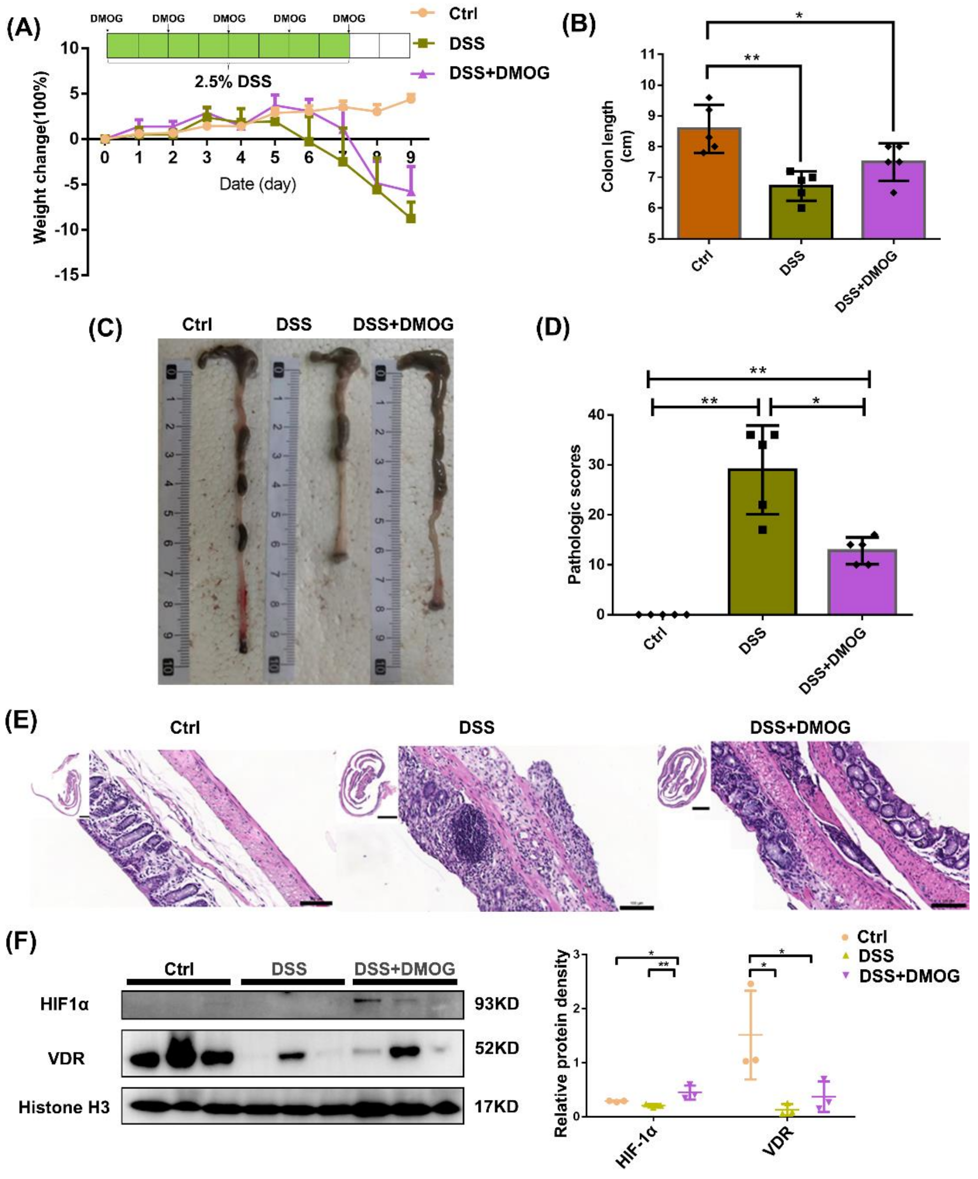

Supplementary Figure 3. DMOG relieved the DSS-induced colitis in mice

Supplementary Figure 3A. The body weight change of control group (Ctrl), DSS colitis group (DSS), and colitis treated with DMOG group (DSS + DMOG). DSS group and DSS+DMOG group receive 2.5% DSS in drinking water for 7 days (showed as green). On days 0, 2, 4, 6 and 8, the DSS + DMOG group was given DMOG i.p. (8 mg in 0.5 ml saline) while DSS group were injected with sterile saline (0.5 ml) at the same intervals (n=5). Supplementary Figure 3B-C: Length of the colon at the end of the experiment in each group. (***P* < 0.01, **P*

< 0.05). Supplementary Figure 3D-E: The HE staining of the colon and their pathological scores in each group. (**$P < 0.01$, *$P < 0.05$). Bars=2000μm or 100μm. Supplementary Figure 3F:VDR and HIF1α protein expression by Western blotting analysis in each group. (**$P < 0.01$, *$P < 0.05$).
